# Integration of the Pokeweed miRNA and mRNA Transcriptomes Reveals Targeting of Jasmonic Acid-Responsive Genes

**DOI:** 10.3389/fpls.2018.00589

**Published:** 2018-05-03

**Authors:** Kira C. M. Neller, Alexander Klenov, Juan C. Guzman, Katalin A. Hudak

**Affiliations:** ^1^Department of Biology, York University, Toronto, ON, Canada; ^2^Department of Electrical Engineering and Computer Science, York University, Toronto, ON, Canada

**Keywords:** jasmonic acid, miRNA, *Phytolacca americana*, plant defense, pokeweed, small RNA, transcriptome

## Abstract

The American pokeweed plant, *Phytolacca americana*, displays broad-spectrum resistance to plant viruses and is a heavy metal hyperaccumulator. However, little is known about the regulation of biotic and abiotic stress responses in this non-model plant. To investigate the control of miRNAs in gene expression, we sequenced the small RNA transcriptome of pokeweed treated with jasmonic acid (JA), a hormone that mediates pathogen defense and stress tolerance. We predicted 145 miRNAs responsive to JA, most of which were unique to pokeweed. These miRNAs were low in abundance and condition-specific, with discrete expression change. Integration of paired mRNA-Seq expression data enabled us to identify correlated, novel JA-responsive targets that mediate hormone biosynthesis, signal transduction, and pathogen defense. The expression of approximately half the pairs was positively correlated, an uncommon finding that we functionally validated by mRNA cleavage. Importantly, we report that a pokeweed-specific miRNA targets the transcript of *OPR3*, novel evidence that a miRNA regulates a JA biosynthesis enzyme. This first large-scale small RNA study of a Phytolaccaceae family member shows that miRNA-mediated control is a significant component of the JA response, associated with widespread changes in expression of genes required for stress adaptation.

## Introduction

The American pokeweed, *Phytolacca americana*, is a non-model plant with promising applications in agriculture. Pokeweed synthesizes pokeweed antiviral protein (PAP), a ribosome inactivating protein with RNA *N*-glycosidase activity (Endo et al., 1988). Several mutants of PAP have been generated and expressed heterologously in transgenic plants to impart novel antiviral and antifungal properties (Zoubenko et al., 1997, 2000; Wang et al., 1998; Dai et al., 2003). Additionally, pokeweed is a heavy metal hyperaccumulator with a potential role in phytoremediation (Peng et al., 2008; Liu et al., 2010; Zhao et al., 2011).

We recently assembled and annotated the pokeweed mRNA transcriptome and reported its regulation by jasmonic acid (JA; Neller et al., 2016). Jasmonates are well-characterized plant signal molecules of the oxylipin family that are involved in abiotic and biotic stress responses, as well as growth and development. JA mediates tolerance to drought, salt, heat and cold stresses, resistance to pathogens and insects, and induces embryogenesis, flowering, and senescence (reviewed in Wasternack and Feussner, 2017). JA is also implicated in tripartite interactions between insect vectors, viruses, and plants (Sun et al., 2017). Interestingly, JA treatment reduces heavy metal-induced oxidative stress (Singh and Shah, 2014) and conversely, heavy metal exposure is associated with biosynthesis of a JA precursor (Foroughi et al., 2014). Given that pokeweed exhibits broad-spectrum pathogen resistance, is a heavy metal hyperaccumulator, and undergoes marked transcriptional reprograming upon JA treatment, we are interested in exploring the range and regulation of defense strategies in this non-model plant.

JA biosynthesis is described by the octadecanoid pathway (reviewed in Schaller et al., 2000). Briefly, alpha-linolenic acid released from plastid membranes is oxidized by a13-lipoxygenase (LOX), followed by coupled dehydration-cyclization by allene oxide synthase (AOS) and allene oxide cyclase (AOC) to generate 12-oxophytodienoic acid (OPDA). This undergoes reduction by OPDA reductase 3 (OPR3) and is followed by three rounds of beta oxidation to form JA. The JA-amido synthetase JAR1 produces the bioactive form of JA, (+)-7-iso-JAIle (JA-Ile); this is perceived by the COI1-JAZ co-receptor and results in ubiquitin-mediated degradation of JAZ repressors and activation of several transcription factors (reviewed in Chini et al., 2016).

Here, we complement our previous transcriptome analysis with a paired RNA-Seq study investigating miRNA-associated regulation of the JA response. miRNAs are non-coding RNAs of ~20–24 nucleotides (nt) that regulate post-transcriptional gene expression (reviewed in Yu et al., 2017). miRNA biogenesis begins with transcription of the *MIR* gene by RNA polymerase II to yield a primary miRNA (pri-miRNA), containing a stem-loop region flanked by unstructured arms. In sequential steps, dicer-like 1 RNase (DCL1) excises the stem-loop to form the pre-miRNA, then produces a smaller duplex comprising the miRNA and its opposing strand, classically termed miRNA^*^. In the cytoplasm, the miRNA associates with argonaute 1 (AGO1) to establish the RNA-induced silencing complex (RISC). Plant miRNAs act primarily through target mRNA cleavage, owing to high miRNA/target sequence complementarity, although translational inhibition has also been observed. miRNAs have been implicated in numerous abiotic and biotic stress responses, such as those induced by temperature, salinity, drought, light, nutrient deficiencies, oxidative stress, and pathogens (reviewed in Khraiwesh et al., 2012; Li et al., 2017; Islam et al., 2018). Previous reports have characterized the effect of JA or its methyl ester (JA-Me) on miRNAs in *Arabidopsis, Nicotiana attenuata, Taxus chinensis, Lycoris aurea*, and *Catharanthus roseus* (Qiu et al., 2009; Bozorov et al., 2012; Zhang et al., 2012; Xu et al., 2016; Shen et al., 2017). Control of JA biosynthesis through miR319-targeted *TCP* transcription factors has also been established (Schommer et al., 2008; Zhao et al., 2015). However, despite the importance of JA in plant development and stress resistance, the expression of JA-responsive miRNAs and targets has not been correlated on a genome-wide scale.

Although a Phytolaccaceae family reference genome is unavailable, current bioinformatic tools support miRNA analysis based on an assembled transcriptome (Dai and Zhao, 2011; Yang and Li, 2011). Here, we identify JA-responsive miRNAs, their potential roles, and construct a miRNA/target interaction network that incorporates correlated expression data from biological replicates. Our results indicate that many defense-related genes are regulated by JA-induced miRNAs, including the JA biosynthesis gene *OPR3*. These novel targets have potential agricultural applications to improve resistance to pathogen and environmental stresses.

## Materials and methods

### Plant growth conditions and jasmonic acid treatment

Pokeweed plants at the 4-leaf stage of growth were sprayed with 5 mM JA suspended in 0.5% ethanol, or 0.5% ethanol alone (control), and leaf tissue was harvested 24 h post treatment (Neller et al., 2016). Through time-course analysis, we established previously that PAP is expressed maximally 24 h following JA treatment (Klenov et al., 2016). This time point was chosen for both the mRNA transcriptome-wide analysis of pokeweed (Neller et al., 2016) and the current paired miRNA analysis, as we were interested in identifying defense genes co-expressed with PAP.

### Small RNA sequencing and data processing

Total RNA was extracted from leaf tissue of pokeweed plants as reported in Neller et al. (2016) and the small RNA fraction was sequenced according to Klenov et al. (2016). Briefly, the 15–30 nt size RNA portion was gel purified and ligated to directional 5p and 3p primers. Following cDNA synthesis and PCR-amplification, the small RNA libraries were sequenced on a SOLiD 5500 XL machine. Six libraries were sequenced, with three biological replicates per treatment. Each replicate consisted of an equal amount of small RNA pooled from three independent plants. The raw sequencing reads were processed as described in Klenov et al. (2016). Non-coding RNA (rRNA, tRNA, snRNA, and snoRNA) and mature miRNA sequences were annotated by mapping reads to the sugar beet genome (Dohm et al., 2014) and the plant miRNA database (Zhang et al., 2010; http://bioinformatics.cau.edu.cn/PMRD/). Mapping was performed with Bowtie software (v. 1.0.1; Langmead et al., 2009) and up to three mismatches were allowed in alignments.

### miRNA prediction and annotation

miRNA prediction was performed with miRDeep-P (v. 1.3; Yang and Li, 2011), a modified version of the miRDeep algorithm (Friedländer et al., 2008) that incorporates an adapted scoring system for plant miRNA biogenesis. Non-annotated small RNA sequences as well as those annotated as mature miRNAs were used as input for miRNA prediction. Small RNA sequences were aligned to the pokeweed mRNA transcriptome (Neller et al., 2016) and only perfect, full-length alignments were retained. For each alignment, a potential precursor sequence of 250 nt in length was excised and its secondary structure and minimum free energy were determined with RNAfold from the ViennaRNA package (v. 2.1.7; Lorenz et al., 2011). Mature miRNAs were predicted based on compatibility of the small RNA with the structure of its precursor. As per miRDeep-P notation, if both 5p and 3p miRNA reads were detected then the most abundant strand was labeled as the mature miRNA. The less abundant strand was defined as the star sequence. Precursor structures were visualized with The UEA Small RNA Workbench (Stocks et al., 2012). Following miRNA prediction, BLASTn-short (Altschul et al., 1997) was used to identify conserved sequences (*E* ≤ 0.001) against all mature sequences from the plant miRNA database. Remaining, non-annotated sequences were considered unique to pokeweed.

### miRNA differential expression analysis

Raw sequencing counts of predicted miRNAs were used for differential expression analysis with the Bioconductor package EdgeR (Robinson et al., 2010) in the statistical program R (R Development Core Team, 2011). Prior to analysis, any miRNA with an abundance of less than 1 read per million (RPM) in each group of replicates was removed. Subsequently, libraries were normalized based on their total number of counts, and the common and tag-wise dispersions were estimated with default parameters. An Exact Test was conducted to detect miRNAs with significant differences in expression between control and JA-treated groups (FDR < 0.05). Each treatment group consisted of three biological replicates.

### Prediction of miRNA targets

Predicted miRNAs were aligned to the pokeweed mRNA transcriptome and putative targets were identified using the Plant Small RNA Target Analysis Server with default parameters (Dai and Zhao, 2011; http://plantgrn.noble.org/psRNATarget/). This program predicts miRNA/target pairs based on reverse complementary matching and target site accessibility, incorporating plant-specific features of target recognition.

### Correlation of miRNA/target expression

A paired design was employed for small RNA and mRNA sequencing; that is, both small RNA and mRNA fractions were sequenced for each biological replicate of each treatment group. The Pearson correlation coefficient (PCC) of each miRNA/target pair was calculated from normalized expression data in RPM and fragments per kilobase per transcript per million mapped reads (FPKM), respectively. Transcript quantification and differential expression results of miRNA targets were obtained from Neller et al. (2016).

### Functional analysis of miRNA targets

Annotation information of miRNA targets was obtained from Neller et al. (2016). Gene ontology (GO) term distribution and enrichment analysis were conducted with Blast2GO software (Conesa et al., 2005). KOBAS 2.0 (Xie et al., 2011) was used to identify enriched KEGG pathways. For enrichment analysis, the test set comprised targets with highly correlated miRNAs (PCC > |0.8|), for which both the target and miRNA were differentially expressed (FDR < 0.05), and the reference set consisted of all miRNA targets.

### Construction of a miRNA/target interaction network

Cytoscape software (v. 3.3.0; Shannon et al., 2003) was used to generate a network of highly correlated miRNA/target pairs (PCC > |0.8|), for which both members were differentially expressed (FDR < 0.05).

### Quantitative reverse transcription PCR (qRT-PCR)

Reverse transcription was performed by combining 250 ng of total pokeweed RNA with 1 μL of 10 μM miRNA-specific stem-loop reverse primer and 1 μL of 10 mM dNTPs in a total volume of 13 μL. The solution was incubated at 65°C for 10 min before cooling on ice for 3 min. To the mixture was added 4 μL of 5X First Strand Buffer (250 mM Tris-HCl pH 8.3, 375 mM KCl, 15 mM MgCl_2_), 1.38 μL of dH_2_O, 1 μL of 0.1 M DTT, 20 units of Murine RNase Inhibitor, and 25 units of Superscript III reverse transcriptase (Invitrogen). The reaction was incubated at 42°C for 1 h and heat inactivated at 70°C for 20 min.

Following reverse transcription, 6 μL of cDNA was combined with 2 μL of 10 μM forward primer, 2 μL of 10 μM reverse primer, 23 μL of dH_2_O, and 33 μL of 2X Sybr Green Mastermix (ABM). Each reaction was divided into three technical replicates and analyzed in a Qiagen Rotor-gene-Q real time PCR cycler. Ct values were calculated with the ΔΔCt relative quantification method. miR156 was used as an internal control, as it showed stable expression based on RNA-Seq results.

### 5′ rapid amplification of cDNA ends (RACE)

Polyadenylated RNA was isolated with the NEBNext Poly(A) mRNA Magnetic Isolation Module. The method of 5′ RACE was based on the Generacer 5′ RACE kit, omitting phosphatase and decapping steps. To relax secondary structure, 250 ng of pokeweed mRNA and 250 ng of RNA adapter were combined in a total volume of 10 μL and incubated at 65°C for 5 min. After cooling on ice for 2 min, 2 μL of 10X T4 RNA Ligase Buffer (500 mM Tris-HCl pH 7.5, 100 mM MgCl_2_, 10 mM DTT), 2 μL of 10 mM ATP, and 10 units of T4 RNA ligase were added and brought to a final volume of 20 μL. The ligation mixture was incubated at 37°C for 1 h, extracted once with acidic phenol/chloroform, precipitated with 5 μg of linear acrylamide, and re-suspended in RNA storage buffer (22.5 mM DTT, 1 mM sodium citrate pH 6.4). Reverse transcription was performed as described above with poly-d(T) adapter primers, followed by one round of non-specific amplification with forward and reverse adapter primers. To isolate cleavage sites, 1 μL of cDNA was used as template for PCR with adapter forward primers and gene-specific reverse primers 3′ of the putative cleavage site. Following one round of nested PCR, the band of interest was gel-purified, cloned, and sequenced. Five cDNA clones were sequenced per validation.

## Results

### Overview of the pokeweed small RNA transcriptome

A summary of our study is provided in Figure S1. Following quality control processing, a total of 57,171,256 clean reads were obtained from control and JA-treated libraries (Table 1). To identify conserved miRNAs in pokeweed, reads were aligned to mature sequences from the plant miRNA database, with up to three allowable mismatches in alignments. Conserved miRNAs comprised a small percentage (<0.5%) of reads, representing 1,381 unique sequences. Other classes of conserved, non-coding RNA in pokeweed (rRNA, tRNA, snRNA, and snoRNA) were annotated by mapping reads to the sugar beet genome. Sugar beet was selected as a reference since it is the closest related species with a sequenced genome. At most, the combination of these remaining non-coding RNA species accounted for 14% of reads. Mapping rates to the pokeweed mRNA transcriptome averaged 18 and 23% for control and JA libraries, respectively. Although relatively low, these rates agree with the finding that most small RNAs were 24-mer heterochromatic siRNAs (59 and 55% for control and JA-treated libraries, respectively; Figure S2). Small RNAs of this class are derived from non-polyadenylated precursors, so these reads were not expected to align to the mRNA transcriptome. Taken together, most of the pokeweed small RNA transcriptome remains un-annotated when compared with sequences from other plants. This indicates that the Phytolaccaceae family represents a source of novel small RNAs and corresponding targets.

**Table 1:** Annotation of small RNA sequences in pokeweed.

**Category**	**C1**	**C2**	**C3**	**JA1**	**JA2**	**JA3**
clean reads	12,794,660	8,560,268	8,109,969	9,697,656	7,997,366	10,011,337
	(100%)	(100%)	(100%)	(100%)	(100%)	(100%)
miRNA	28,149	28,249	28,385	32,002	25,592	34,039
	(0.22%)	(0.33%)	(0.35%)	(0.33%)	(0.32%)	(0.34%)
rRNA	220,069	111,283	97,320	180,376	110,364	218,247
	(1.72%)	(1.30%)	(1.20%)	(1.86%)	(1.38%)	(2.18%)
tRNA	319,867	215,719	223,835	662,350	570,212	1,098,243
	(2.50%)	(2.52%)	(2.76%)	(6.83%)	(7.13%)	(10.97%)
snRNA	6,141	3,339	2,436	5,830	4,798	6,028
	(0.05%)	(0.04%)	(0.03%)	(0.06%)	(0.06%)	(0.06%)
snoRNA	6,653	3,510	2,430	5,808	5,598	5,986
	(0.05%)	(0.04%)	(0.03%)	(0.06%)	(0.07%)	(0.06%)
mRNA transcriptome	2,566,606	1,687,229	1,428,977	2,175,184	1,848,191	2,474,803
	(20.06%)	(19.71%)	(17.62%)	(22.43%)	(23.11%)	(24.72%)
unannot.	9,647,174	6,510,940	6,326,586	6,636,106	5,432,611	6,173,991
	(75.40%)	(76.06%)	(78.01%)	(68.43%)	(67.93%)	(61.67%)

*The small RNA fraction was sequenced from leaf tissue of 4-leaf plants treated with ethanol (C, control) or jasmonic acid (JA). Clean reads were mapped to the sugar beet genome and mature miRNA sequences from the plant miRNA database, with three allowable mismatches, and to the pokeweed mRNA transcriptome, with perfect alignment. Three biological replicates were sequenced per treatment*.

### miRNA prediction and differential expression analysis

Pokeweed miRNAs were predicted computationally with miRDeep-P, a plant-specific program that incorporates principles of miRNA biogenesis. Briefly, unannotated reads and those annotated as mature miRNA sequences were aligned to the pokeweed mRNA transcriptome to identify likely miRNA precursors. For each alignment, a flanking region of mRNA was extracted and its secondary structure predicted. Mature miRNAs were identified based on the likelihood that a particular small RNA sequence originated from the biological processing of a miRNA precursor. This strategy led to the prediction of 582 miRNAs in pokeweed; their sequences are provided in Table S1. To identify which of these miRNAs were conserved, a BLASTn-short search was conducted against mature sequences from the plant miRNA database (*E*-value ≤ 0.001). A total of 24 conserved miRNAs were annotated (Table 2). Among these, the most abundant were miR156, miR169, miR535, and miR396.

**Table 2:** Conserved miRNAs in pokeweed.

**miRNA**	**Sequence (5′-3′)**	**Abundance (RPM)**
156	UGACAGAAGAGAGUGAGCAC	2, 836.34
157	GCUCUCUAUGCUUCUGUCAUC	9.29
159	GAUCAUGUGGUAGCUUCACC	203.98
162	UCGAUAAACCUCUGCAUCCAG	121.20
164	UGGAGAAGCAGGGCACGUGCA	531.04
166	UCGGACCAGGCUUCAUUCCUC	16.09
166	CCGGACCAGGCUUCAUUCCCC	14.78
166	UCGGACCAGGCUUCAUUCCCC	11.21
167	UGAAGCUGCCAGCAUGAUCUG	182.36
168	UCGCUUGGUGCAGGUCGGGAA	94.33
169	CAGCCAAGGAUGACUUGCCGG	2126.80
169	UAGCCAAGGAUGACUUGCCUG	131.25
171	UGAUUGAGCCGUGCCAAUAUC	412.17
171	UGAUUGAGCCGCGCCAAUAUC	193.00
172	CGAAUCUUGAUGAUGCUGCAU	69.79
319	UUUGGAUUGAAGGGAGCUC	9.66
394	UUUGGCAUUCUGUCUACCUCC	2.57
395	CUGAAGUGUUUGGGGGAACUC	7.24
396	UUCCACAGCUUUCUUGAACUG	1897.91
396	UUCCACAGCUUUCUUGAACUU	885.08
403	UUAGAUUCACGCACAAACUCG	141.05
535	UGACGAUGAGAGAGAGCACGC	1991.70
3954	UUAGACAGAGAAAUCACGGUUG	49.85
5203	AGUGACAGAUAUUAUGGACCGGAG	0.26

*Following miRNA prediction, conserved sequences were identified through BLASTn-short alignment (E ≤ 0.001) against the plant miRNA database. The average abundance of each miRNA is provided in reads per million (RPM)*.

**Figure 1:**
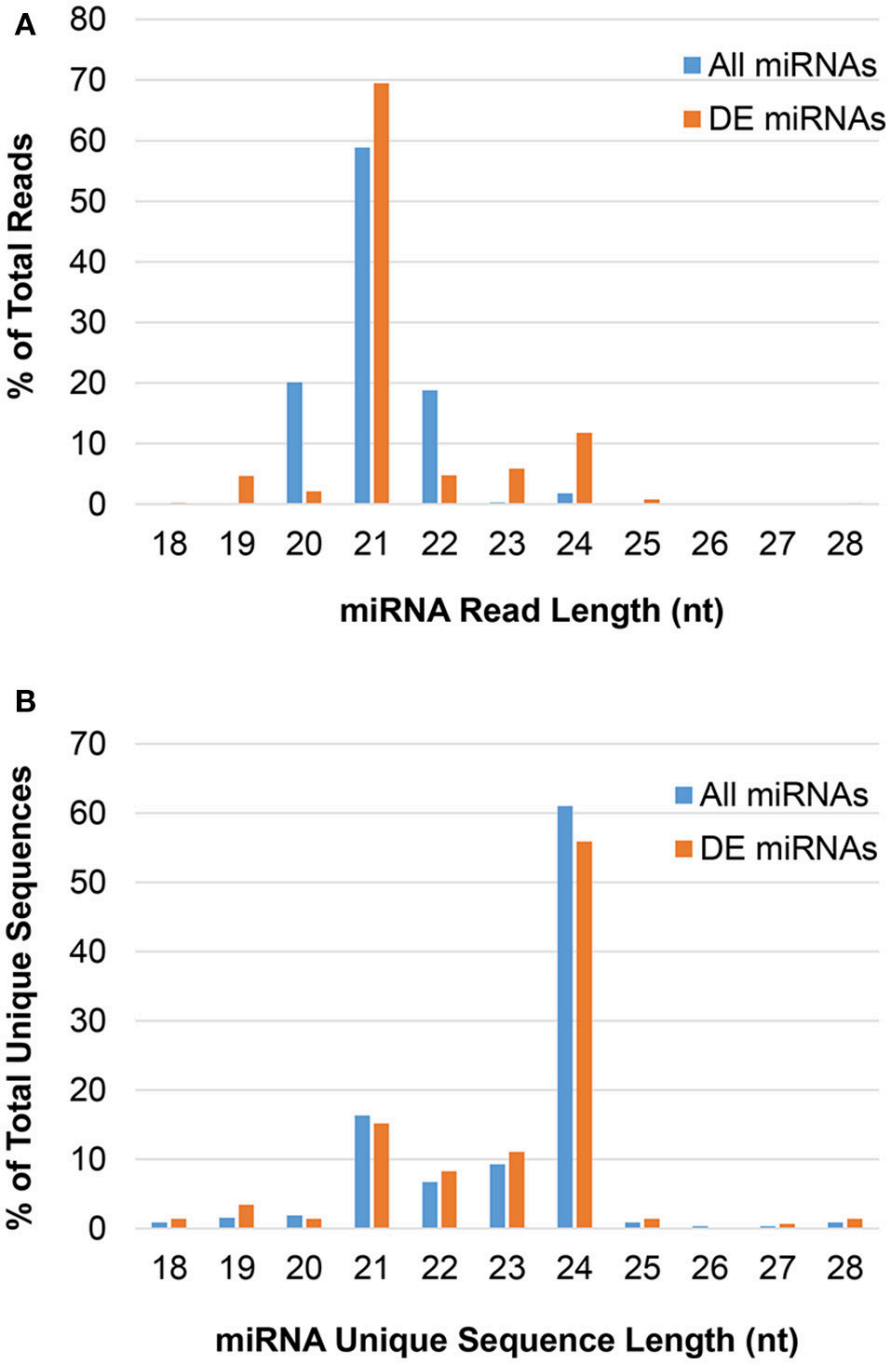
The length distribution of predicted miRNAs in pokeweed. **(A)** Reads; **(B)** unique sequences. DE, differentially expressed; nt, nucleotides.

The majority of predicted miRNAs were 21-nt, as expected (Figure 1A); however, this pool had low sequence diversity, as many 21-nt reads had the same sequence (Figure 1B). Conversely, non-canonical 24-nt miRNAs had high sequence diversity and likely targeted numerous different genes, even though they comprised a small fraction of the miRNA pool. These trends were consistent following differential expression analysis to identify JA-responsive miRNAs. That is, the 24-nt miRNAs contained the greatest number of unique sequences in both the total miRNA and JA-responsive miRNA pools.

**Figure 2:**
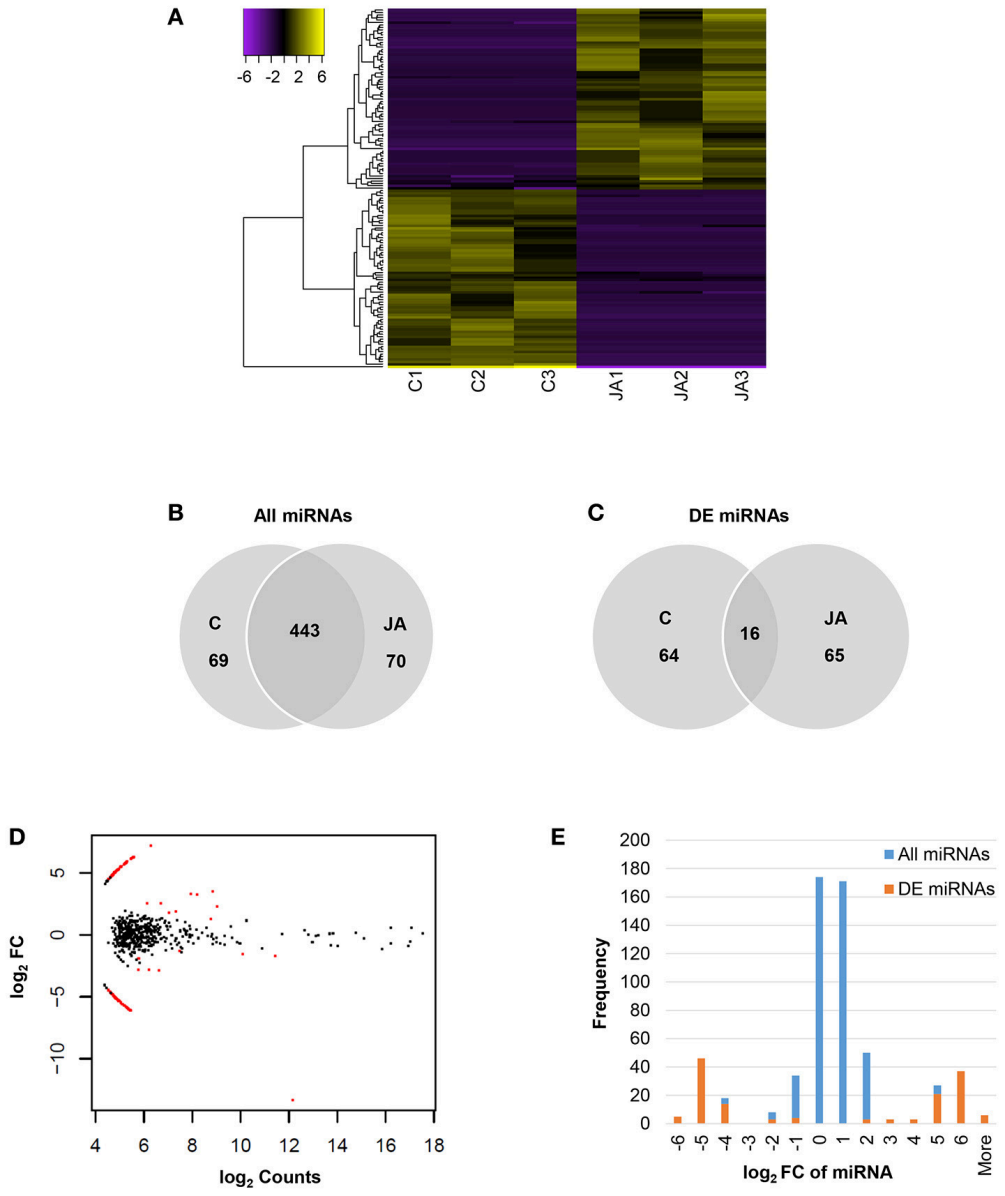
Identification of JA-responsive miRNAs in pokeweed. **(A)** Heat map of expression values (log_2_RPM, median-centered) of differentially expressed miRNAs (FDR < 0.05). **(B,C)** Depict treatment-specific expression patterns of all miRNAs and differentially expressed miRNAs, respectively. **(D)** MA plot indicating all differentially expressed miRNAs (red). **(E)** Histogram of miRNA fold change; x-axis values indicate upper bin numbers. DE, differentially expressed; FC, fold change.

Of 582 miRNAs, 145 showed significant changes in abundance with JA treatment, relative to control (FDR < 0.05; Figure 2A and Table S2). Among these were two conserved miRNAs, miR172 and miR395, with log_2_ fold changes of −13.4 and 2.30, respectively. The numbers of up and down-regulated miRNAs were equal, with 73 and 72 sequences, respectively. Interestingly, although a majority, 443 of the 582 miRNAs, were present in both control and JA samples (Figure 2B), most differentially expressed miRNAs were condition-specific (Figure 2C). That is, most were detected in either all control replicates or all treated replicates; only 16 miRNAs (11% of those differentially expressed) were found in all samples. Furthermore, most JA-responsive miRNAs were of low abundance and displayed high fold changes within a narrow range (Figure 2D). We observed a symmetrical distribution of miRNA fold changes (Figure 2E). Of all differentially expressed miRNAs, those that decreased in abundance with JA had an average log_2_FC and standard deviation of −5.13 ± 1.38, while those that increased with JA had values of 4.92 ± 1.07. Therefore, JA treatment alters sequence diversity within the miRNA pool, and discrete fold changes in expression of JA-responsive miRNAs may be required for biological effect.

### Identification of JA-responsive miRNA/target pairs

We used the Plant Small RNA Target Analysis Server to identify pokeweed mRNA transcripts having high sequence complementarity with the 582 predicted miRNAs, which resulted in a total of 25,931 miRNA/target pairs. To identify the most biologically relevant interactions, the list was filtered to include only JA-responsive miRNAs and targets (FDR < 0.05), resulting in a subset of 516 miRNA/target pairs. That is, for each pair, both miRNA and target were differentially expressed with JA. The rationale for limiting our analysis in this manner was to include only interactions that could potentially mediate a functional response in the plant through the alteration of mRNA abundance, given that plant miRNAs act predominately through target mRNA cleavage.

**Figure 3:**
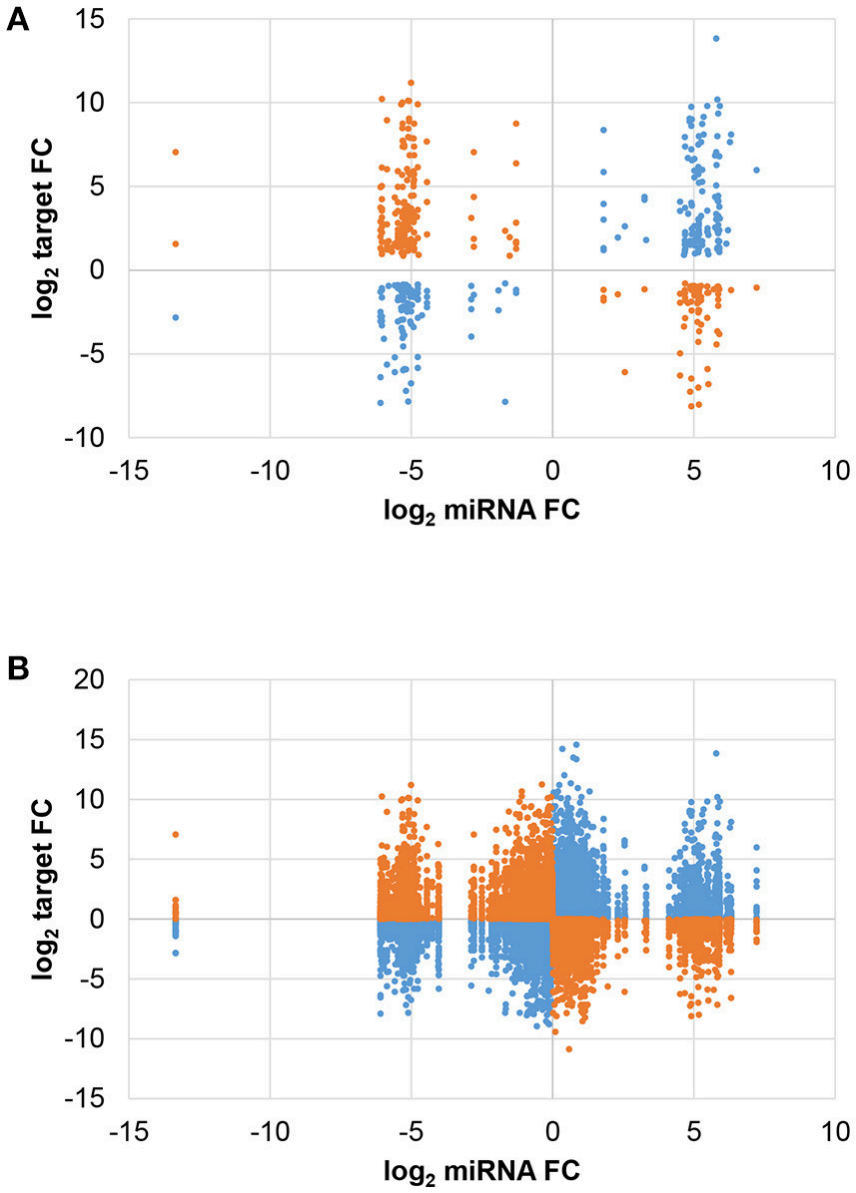
Comparison of miRNA and target expression changes. The fold change (FC) of each miRNA was plotted against that of its predicted target. Blue and orange indicate positive and negative correlations, respectively, of miRNA/target expression changes. **(A)** Differentially expressed miRNAs and targets (FDR < 0.05). **(B)** All miRNAs and targets.

The expression change of each miRNA and target forming the 516 JA-responsive pairs is shown in Figure 3A. The number of negative and positive miRNA/target expression correlations was similar, with 274 pairs showing opposite JA-induced changes (i.e. miRNA ↑ and target ↓ or vice versa) and 242 pairs with parallel changes (i.e. miRNA and target both ↑ or ↓). Most strikingly, while miRNA expression change was limited to a narrow range, target change was much more variable. Interestingly, plotting all miRNA/target pairs revealed that the non-random distribution was inherent in the overall miRNA population, as shown by trimodal clustering (Figure 3B). These results indicate that JA treatment induces a certain threshold of miRNA expression change, for those miRNAs that are JA-responsive, suggesting an optimal miRNA fold change for effective JA control.

This RNA-Seq study incorporated paired small RNA and mRNA samples; that is, each RNA sample was sequenced to quantify both small RNA and mRNA levels. This paired design, combined with the use of independent biological replicates, enabled the identification of miRNA/target pairs with highly correlated expression. The PCC was calculated for each of the 516 miRNA/target pairs described above and the distribution of correlations is shown in Figure S3. There was a clear enrichment in moderate and high correlations; this was expected as differential expression analysis and sequence complementarity filters had already been applied to retain the most likely miRNA/target candidates. Specifically, we were interested in highly correlated pairs, defined as those with PCC > |0.8|. This cut-off reduced the number of pairs from 516 to 171, comprising 97 positively correlated pairs and 74 negatively correlated ones. These 171 pairs are most biologically relevant since the expression of each miRNA is associated with the expression of its target.

### Functional analysis of highly correlated miRNA/target pairs

Following the identification of highly correlated miRNA/target pairs, our next goal was to functionally characterize them. Target annotations were obtained from our previous study of the pokeweed mRNA transcriptome (Table S3). Based on KEGG pathway enrichment, the top pathways were associated with plant stress and defense responses, including “peroxisome,” “alpha-Linolenic acid metabolism,” and “plant-pathogen interaction” (Table 3). The corresponding targets mapping to each enriched pathway revealed key genes that may be regulated by miRNAs. Notably, these included two genes associated with JA biosynthesis (*OPR3* and *AOC3*) as well as an integrator of JA and ethylene signaling (Ethylene-responsive transcription factor 1B, *ERF1B*). Although miRNA-mediated control of transcription factors involved in JA biosynthesis has been reported, our results indicate that miRNAs directly target genes of the JA biosynthesis pathway.

**Table 3:** Top pathways involving highly correlated, JA-responsive miRNA/target pairs.

**Pathway**	**FDR**	**Annotated genes**
Peroxisome	0.077	2-hydroxyacyl-CoA lyase Peroxisomal nicotinamide adenine dinucleotide carrier Isocitrate dehydrogenase
alpha-Linolenic acid metabolism	0.077	Allene oxide cyclase 3, chloroplastic 12-oxophytodienoate reductase 3
Plant-pathogen interaction	0.077	Calcium-dependent protein kinase 16 Calmodulin-like protein 1
Glutathione metabolism	0.12	Isocitrate dehydrogenase [NADP] Glutathione S-transferase U9
Plant hormone signal transduction	0.13	Ethylene-responsive transcription factor 1B Auxin response factor 1 Two-component response regulator ARR9

*KEGG pathway enrichment was performed relative to all miRNA/target pairs, which served as the reference set. The specific genes annotated in each enriched pathway are indicated*.

Since pokeweed is a non-model plant with limited resources for annotation, some targets could not be mapped to GO terms or KEGG pathways, or their functional associations lacked substantial detail. To account for this, we manually assessed the 171 highly correlated miRNA/target pairs and cross-referenced the literature to find genes associated with plant stress and defense responses. The results are summarized in Table 4, revealing novel targets involved in hormone signaling, hypersensitive response, lignin biosynthesis, and oxidative stress response. Importantly, some miRNAs in Table 4 have more than one target, such as 110629_x73, which is predicted to regulate two genes encoding anti-microbial proteins, and 419489_x11, which targets genes involved in both JA signaling and cellular detoxification. Taken together, it is evident that targets of highly correlated miRNAs contribute to various aspects of biotic and abiotic stress responses.

**Table 4:** Identification of highly correlated, JA-responsive miRNA/target pairs implicated in plant stress and defense responses.

**miRNA**		**Target**			
**ID**	**log_2_FC**	**ID**	**log_2_FC**	**Name**	**Function**
204283_x13	−5.11	c113292_g1_i1	2.82	Allene oxide cyclase 3, chloroplastic	JA biosynthesis (Ziegler et al., 2000)
98462_x2365	−1.69	c19572_g1_i1	2.36	12-oxophytodienoate reductase 3	JA biosynthesis (Schaller et al., 2000)
419489_x11	4.92	c37552_g1_i1	9.01	Ethylene-responsive transcription factor 1B	JA signaling (Lorenzo et al., 2003)
404574_x21	5.83	c58992_g2_i1	8.02	NAC domain-containing protein 72	Abiotic stress-associated transcription factor (Tran et al., 2004)
499371_x14	5.29	c58861_g12_i1	1.51	Auxin response factor 1	Transcription repressor, auxin response (Ellis et al., 2005)
297830_x3990	−13.36	c40819_g1_i1	−2.81	Two-component response regulator ARR9	Negative regulator of cytokinin signaling (To et al., 2004)
430760_x13	5.15	c47336_g1_i1	6.17	Zinc finger protein ZAT10	Transcription repressor, abiotic stress response (Mittler et al., 2006)
144454_x98	1.80	c17159_g1_i1	5.88	Transcription factor bHLH25	JA-induced transcription factor (Heim et al., 2003)
319549_x8	−4.46	c60508_g2_i1	2.14	TGACG-sequence-specific DNA-binding protein TGA-2.1	SA-induced transcription activator (Niggeweg et al., 2000)
225512_x26	−6.05	c52120_g1_i1	2.58	Probable protein phosphatase 2C 73	Negative regulator of ABA signaling (Umezawa et al., 2009)
66102_x232	3.25	c33290_g1_i1	4.22	U-box domain-containing protein 19	Protein ubiquitination; negative regulator of ABA and drought responses (Liu et al., 2011)
513239_x9	4.68	c42333_g1_i1	1.72	F-box protein FBW2	Protein ubiquitination; response to ABA (Earley et al., 2010)
110629_x73	−2.89	c54352_g2_i1	−1.73	Glucan endo-1,3-beta-glucosidase 1	Anti-microbial activity; viral pathogenesis (Beffa et al., 1996)
110629_x73	−2.89	c13510_g1_i1	3.13	Polygalacturonase inhibitor	Anti-microbial activity (Kalunke et al., 2015)
456330_x11	3.90	c80075_g1_i1	4.92	Snakin-2	Anti-microbial activity (Berrocal-Lobo et al., 2002)
443361_x21	5.89	c58046_g10_i1	−3.80	Probable cinnamyl alcohol dehydrogenase 6	lignin biosynthesis (Sibout et al., 2005)
443361_x21	5.89	c23015_g1_i1	1.73	Dirigent protein 21	lignin biosynthesis (Davin and Lewis, 2000)
319549_x8	−4.46	c17278_g1_i2	5.28	Uclacyanin-2	Lignin biosynthesis (Nersissian et al., 1998)
419489_x11	4.92	c57722_g7_i1	2.17	Glutathione S-transferase U9	Cellular detoxification (Wagner et al., 2002)
478663_x13	5.18	c54687_g4_i1	3.06	Isocitrate dehydrogenase [NADP]	Response to oxidative stress (Valderrama et al., 2006)
1213_x8	4.50	c56365_g1_i1	−1.39	Proline-rich receptor-like protein kinase PERK1	Hypersensitive response (Silva and Goring, 2002)
485379_x20	5.78	c59820_g2_i8	3.04	Calcium-dependent protein kinase 16	Hypersensitive response (Coca and San Segundo, 2010)
513239_x9	4.68	c48318_g1_i3	−2.85	Hypersensitive-induced response protein 1	Hypersensitive response (Qi et al., 2011)

*miRNA and Target IDs are formatted per miRDeep and Trinity notation, respectively. Log_2_FC indicates fold change of miRNA and target levels in JA-treated libraries relative to control libraries*.

We constructed an interaction network to visualize the 171 highly correlated, differentially expressed miRNA/target pairs (Figure S4). The network includes 67 unique miRNAs, the majority of which (40) had more than one target. Major hubs are located toward the top of the network; these are cases where a single miRNA has many targets, and different hubs are often connected by a common target. Enrichment analysis was conducted to identify overrepresented GO terms associated with major hubs; however, no significant terms were found. This suggests that though individual miRNA/target pairs are involved in plant defense, these miRNAs also control different mRNAs with diverse functions, rather than all targets of a single miRNA being involved in a single response (Table 3).

Precursor structures of the 67 miRNAs are provided in Figure S5. Although variability was evident in terms of overall precursor shape and positioning of mature sequence, the mature miRNA tended to be in the middle or upper stem of a stable hairpin structure. We did note a few unexpected cases wherein the mature miRNA was mostly or completely contained within a non-hairpin loop; these cases may have arisen from limitations in the RNA folding and/or miRNA prediction programs.

### Validation of miRNA differential expression and functional activity

**Figure 4:**
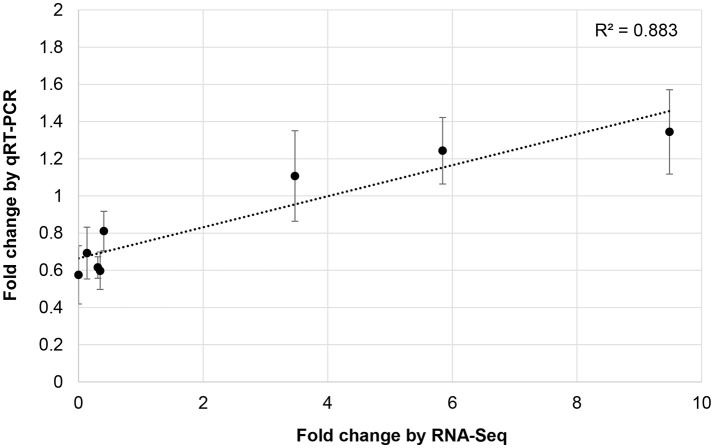
Validation of JA-responsive miRNAs. The correlation of JA-induced expression changes obtained from RNA-Seq and qRT-PCR is shown for 8 miRNAs. Results for qRT-PCR represent the mean from two or three independent biological replicates for each miRNA. Bars indicate the standard error associated with qRT-PCR.

**Figure 5:**
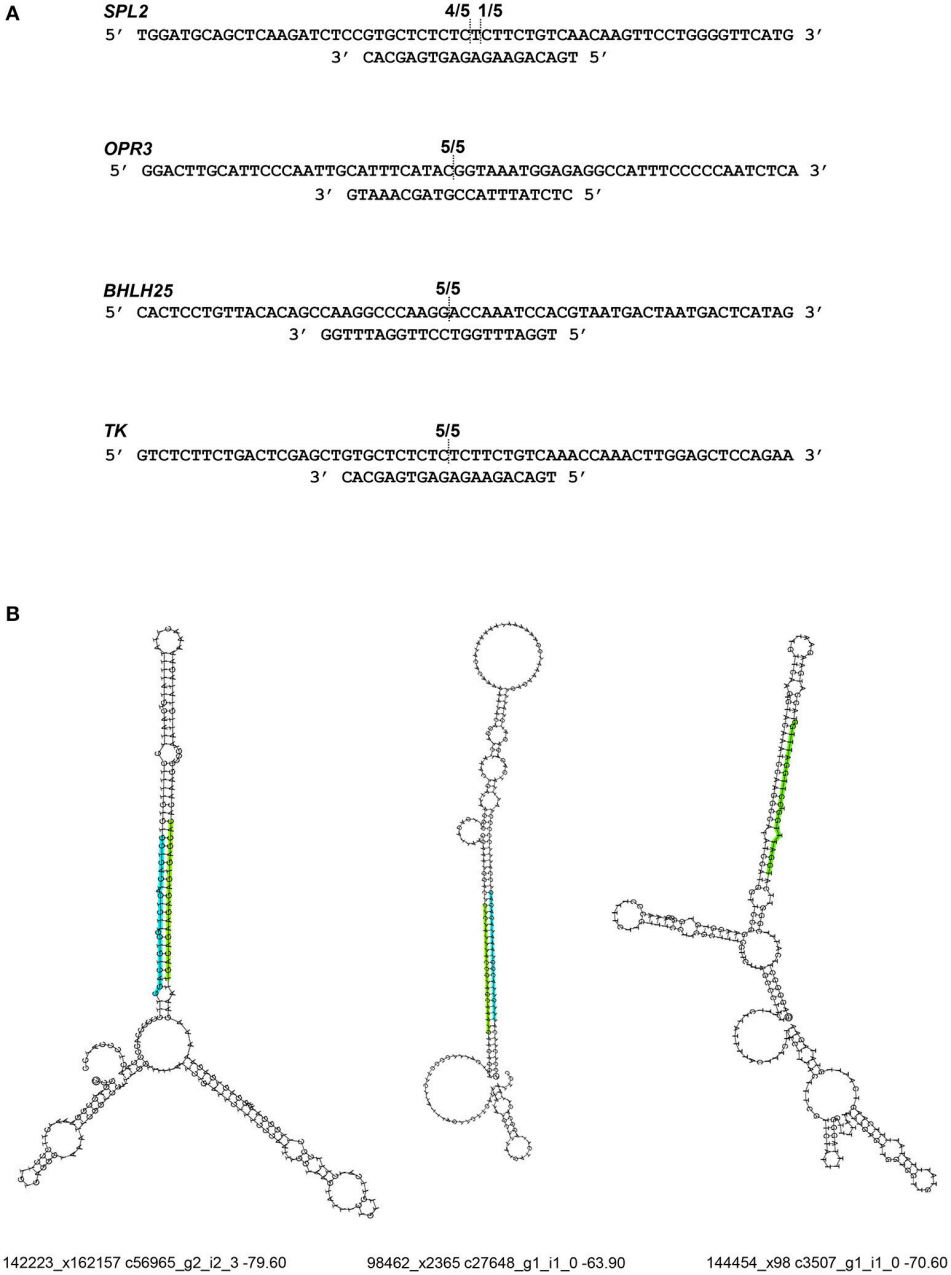
Validation of miRNA target cleavage. **(A)** miRNA-induced cleavage of mRNA targets was validated through 5′RACE. The upper sequence indicates the mRNA target and the lower sequence is its miRNA. Fractions indicate cleavage sites from individual cDNA clones. From top to bottom, miRNAs are: miR156, miR98462_x2365, miR144454_x98, and miR156. **(B)** Precursor structures are shown for each miRNA. The mature miRNA sequence is indicated in green. If a miRNA star strand was detected, it was indicated in blue. For each structure, the corresponding miRNA ID, precursor mRNA ID, and minimum free energy are provided.

To validate RNA-Seq results, we measured the expression of eight miRNAs (98462_x2365, 297830_x3990, 66102_x232, 110629_x73, 114897_x905, 144454_x98, 62255_x144, 50891_x48) by qRT-PCR from control and JA-treated plants (Figure 4). These miRNAs were selected to represent a range of fold changes (9.5 × 10^−5^ to 9.5) and abundances (0.4–44 RPM), and all had a highly correlated target that was functionally annotated through BLASTx. The R^2^ correlation value was 0.88, indicating high correspondence between the two methods of differential expression analysis. The fold-change of transcript levels quantified with RNA-Seq were higher than those quantified with qRT-PCR, as observed previously (Baran-Gale et al., 2015). This difference is likely due to lower amplification efficiency of stem-loop primers used in qRT-PCR, compared to adapter-specific primers used for RNA-Seq. To verify miRNA-induced cleavage of a specific mRNA, four targets were validated by 5′RACE: c56431_g2_i2 (squamosa promoter-binding-like protein 2*, SPL2*), c59355_g7_i1 (thymidine kinase*, TK*), c19572_g1_i1 (*OPR3)*, and c17159_g1_i1 (transcription factor bHLH25*, BHLH25)* (Figure 5A). The *SPL2*/miR156 pair was selected as a positive control, as it is a well-established interaction. mRNA cleavage was detected at the expected site, between the 10th and 11th nucleotide of the miRNA binding site, in all cases except for *OPR3*, where cleavage occurred between the 11th and 12th nucleotide. *OPR3* and *BHLH25* were targeted by pokeweed-specific miRNAs (98462_x2365 and 144454_x98, respectively) and represent two confirmed interactions from Table 4; both miRNAs were also validated through qRT-PCR (above). *TK* was identified unexpectedly as a novel target of the well-conserved miR156 and was significantly JA-responsive based on mRNA-Seq results. Precursor structures of miR156 and the two novel miRNAs are shown in Figure 5B. Both miR156 and miR 98462_x2365 had detectable star sequences while 144454_x98 did not, likely owing to its lower abundance. All three precursors adopted stable hairpin formations.

## Discussion

We have performed a large-scale miRNA analysis of pokeweed, with emphasis on JA-induced genes that contribute to biotic and abiotic stress tolerance. A comprehensive resource of 57,171,256 high-quality small RNA reads has been generated, representing the first available small RNA transcriptome of any Phytolaccaceae family member. Through miRNA prediction and annotation, we identified conserved and novel miRNAs in pokeweed. Differential expression analysis revealed that the majority of JA-responsive miRNAs were low in abundance, condition-specific, and exhibited a narrow range of expression change. Our paired RNA-Seq experimental design allowed the prediction of high-confidence miRNA targets and construction of an interaction network to visualize the global connection between miRNAs and their different targets.

### Pokeweed miRNAs have low sequence conservation with those of other plants

Unexpectedly, less than 0.5% of pokeweed small RNA reads represented conserved miRNAs, based on comparison with available plant sequences. Compared with other primary studies of non-model plants, this number seems low. For example, in black pepper, 11% of small RNA reads were conserved miRNAs, with up to two mismatches in alignments (Asha et al., 2016). In *Vriesea carinata*, 16% of small RNA reads were conserved miRNAs, with only perfect alignments permitted (Guzman et al., 2013). Since this is the first miRNA study of any Phytolaccaceae member, low sequence conservation in pokeweed may be a result of species specificity. Although the majority of plant miRNAs are species-specific (Chávez Montes et al., 2014), the low level of conservation in pokeweed is indicative of a relatively unique miRNA pool.

We performed a stringent miRNA annotation by requiring precursor evidence in addition to mature sequence conservation. Using this strategy, 24 conserved miRNAs were identified in pokeweed, and their expression levels were similar to those observed in other plants (Chávez Montes et al., 2014). Although 1,381 sequences were conserved, a miRNA precursor was predicted for only 1.6%. Others have reported similar findings. For example, in black pepper, 33,350 miRNA sequences were conserved but precursors were found for only 50, or 0.15% (Asha et al., 2016). Despite the common strategy of identifying miRNAs based on sequence conservation alone, our analysis indicates that this method may overestimate the number of true miRNAs.

We also observed non-canonical miRNAs that were 24-nt in length and had high sequence diversity relative to 21-mers. Similar findings have been reported by others (Wan et al., 2012; Guzman et al., 2013; Hackenberg et al., 2013; Shuai et al., 2013). Longer miRNAs tend to be species-specific and are reflective of newly evolved “proto-miRNAs” that undergo imprecise processing by DCL enzymes; interestingly, 24-nt miRNAs can enter the heterochromatic siRNA pathway to direct chromatin modifications of target genes (Axtell, 2013).

### JA treatment induces defined changes in the miRNA transcriptome

Differential expression analysis revealed that JA-responsive miRNAs tended to be condition-specific and low in abundance, with narrowly clustered expression patterns. Condition specificity and low abundance may indicate that, at certain times, sequence diversity of the miRNA pool is more biologically relevant than miRNA abundance. For example, low abundant miRNAs may be early responders to stress. In support of this idea, Pandey et al. (2008) reported only 2.4% overlap between the small RNA populations of untreated *Nicotiana attenuata* plants and those having undergone 45 minutes of insect elicitation, which strongly upregulated the JA response. Since low-abundant miRNAs tend to be species-specific (Chávez Montes et al., 2014), analysis of corresponding targets may lead to the identification of key genes contributing to distinctive traits.

Our expression results suggest that a certain threshold of miRNA change is necessary to mediate an effective response to JA. Specifically, log_2_FC values of JA-responsive miRNAs clustered around +5 and −5. JA-associated thresholding is further supported by the finding that log_2_FC values of insect-induced small RNAs in *N. attenuata* ranged from +4 to −4 (Pandey et al., 2008). Clustering of miRNAs has also been reported. In cotton under salt stress, Peng et al. (2014) observed four discrete groups when the expression of salt-responsive miRNAs was plotted against that of targets; such clustering existed for both positively and negatively correlated pairs. These patterns may reflect co-regulated miRNA expression owing to control by common transcription factors, which are themselves differentially expressed.

Although we identified many JA-responsive miRNAs, only two were conserved with other plants: miR172 and miR395, which decreased and increased with JA, respectively. In contrast, others have reported several conserved, JA-responsive miRNAs (Qiu et al., 2009; Bozorov et al., 2012; Zhang et al., 2012; Xu et al., 2016; Shen et al., 2017). One reason for this discrepancy may be our stringent requirements for differential expression, owing to the availability of biological replicates. Other important differences between our work and that cited above include the use of plants deficient in JA biosynthesis, as well as the investigation of cell cultures rather than whole plants. Although miR172 was reported as JA-responsive by others, its magnitude and direction of change differed across studies. In contrast, miR395 has not been reported as JA-responsive; its most well-established role to date is in sulfur assimilation (Matthewman et al., 2012). Interestingly, miR395 increased in *Arabidopsis* after heavy metal treatment and contributed to cadmium detoxification in *Brassica napus* (Jagadeeswaran et al., 2013; Zhang et al., 2013). Given that heavy metal stress is associated with biosynthesis of a JA precursor (Foroughi et al., 2014), and JA treatment reduces heavy metal stress (Singh and Shah, 2014), there is a likely association between miR395 and JA.

### JA-responsive miRNAs are both positively and negatively correlated with targets

To identify the top miRNA/target candidates, three factors were considered: differential expression, sequence complementarity, and correlation analysis. Pearson correlation is widely used to infer miRNA/target relationships (Muniategui et al., 2013). However, since high correlation does not necessarily indicate causation, we applied this filter after first considering more biologically relevant parameters. Although there is a tendency in literature to report only pairs with negatively correlated expression, we observed a relatively equal distribution of positive and negative correlations. This finding has been observed in other plant studies, reinforcing the concept that both dynamics are prevalent and worthy of consideration (Lopez-Gomollon et al., 2012; Peng et al., 2014; Wen et al., 2016). Positive correlations between the expression of a miRNA and its target can arise from miRNA-mediated spatial restriction of a target (Kidner and Martienssen, 2004; Nikovics et al., 2006; Levine et al., 2007; Kawashima et al., 2009). In *Arabidopsis* roots, a positive temporal and negative spatial correlation was found for the expression of miR395 and its target, sulfur transporter *SULTR2;1*. Authors hypothesized that under sulfur starvation, phloem-specific co-expression of miR395/*SULTR2;1* restricts expression of *SULTR2;1* to the xylem (Kawashima et al., 2009). As another example, the extent of leaf serration in *Arabidopsis* is controlled by spatial restriction of MIR164A-targeted *CUC2* (Nikovics et al., 2006).

Additionally, it is thought that miRNAs operate through two independent mechanisms that are not mutually exclusive: expression tuning, whereby they modify the mean target abundance, and expression buffering, in which they reduce the variance of target abundance around a preset mean (Wu et al., 2009). Negative miRNA/target expression correlations could arise from the first mechanism and positive correlations from the second. Expression buffering consists of negative feedback loops and incoherent feed forward loops (Tsang et al., 2007; Wu et al., 2009). Consider a miRNA, “miR” and its target gene, “*T*.” As summarized by Wu et al., in a negative feedback loop, protein T is a positive regulator of miR, such that increased T results in increased miR, dampening oscillations in the expression of either gene. An example of this dynamic has been shown within the miRNA biogenesis pathway itself: miR162 targeting of *DCL1* (Xie et al., 2003). In an incoherent feed forward loop, consider an additional gene, “*A*”, whose product increases levels of both T and miR. Since A regulates the level of T, any change in the level of A would also affect that of T. However, variation in A is also passed to miR, which then buffers the fluctuation in T. An interesting example here is the maintenance of AGO1 homeostasis by (1) miR168 targeting; (2) transcriptional co-expression of miR168 and AGO1; and (3) preferential stabilization of miR168 by AGO1. This example combines two negative feedback loops (1 and 3), with an incoherent feed forward loop (2). Though positive correlations of miRNA and target gene levels are not commonly described, examples exist of their biological relevance.

We validated the miRNA-induced cleavage of a positively correlated target, *BHLH25*. Little is known about the biological role of this transcription factor, but it is reportedly JA-responsive (Heim et al., 2003) and transgenic *Arabidopsis* plants overexpressing this gene have altered root and shoot morphology and greater susceptibility to cyst nematode infection (Jin et al., 2011). Increased expression of the miRNA in tandem with *BHLH25* could buffer levels of this transcription factor during JA induction. For example, an increase in BHLH25 protein could increase the expression of its own miRNA directly (negative feedback loop), and/or an upstream transcription factor could simultaneously increase expression of both BHLH25 and its miRNA (incoherent feed forward loop).

### JA-responsive miRNA/target pairs are associated with multiple levels of plant defense

We identified putative miRNA targets involved in JA biosynthesis and signaling. Two genes encoding JA biosynthesis enzymes were identified, *AOC3* and *OPR3*, and the miRNA-induced cleavage of *OPR3* mRNA was verified by 5′ RACE. *OPR3* and more recently, *OPR2*, were shown to be required for JA biosynthesis in *Arabidopsis* (Stintzi and Browse, 2000; Chini et al., 2018). Although miR319 targets *TCP* transcription factors and indirectly regulates JA biosynthesis (Schommer et al., 2008), this is the first reported miRNA-induced cleavage of a transcript encoding a JA biosynthesis enzyme. Others have predicted complementary miRNA/target pairs comprising JA biosynthesis enzymes upstream in the pathway, namely *LOX2/3* and *AOS*; however, these interactions have not been functionally validated (Pandey et al., 2008). Given that *AOC3* and *OPR3* mRNA abundance increased with JA, while their corresponding miRNAs decreased, such regulation would contribute to the known positive feedback loop of JA biosynthesis (Wasternack and Hause, 2013). Interestingly, although miR319 was identified in pokeweed, it was not JA-responsive. In tomato, JA treatment significantly reduced miR319 expression, which was associated with an increase in *TCP4* mRNA (Zhao et al., 2015). miR319 targets several class II TCPs, which function as transcriptional activators of *LOX2* (Schommer et al., 2008). We hypothesize that the existence of pokeweed-specific, JA-responsive miRNAs reflects an alternative strategy to regulate JA biosynthesis. This hypothesis is further supported by the absence of miR319 binding sites in any TCP-encoding mRNA transcript in pokeweed. We also predicted that *ERF1B* is targeted by a miRNA; the encoded transcription factor is a key mediator of JA signal transduction that also integrates ethylene-induced signals (Wasternack and Hause, 2013). To the best of our knowledge, this is the first report that *OPR3* mRNA is cleaved by a miRNA, and that *AOC3* and *ERF1B* are putative miRNA targets.

Apart from JA biosynthesis and signaling, we identified putative targets having more widespread roles in stress tolerance. These are summarized in Table 4 and include several factors involved in salicylic or abscisic acid (ABA) signaling, with well-established roles in pathogen and environmental stress resistance, and others associated with classic growth hormones. Analysis of the expression dynamics of these targets provides insight into the role of miRNAs in mediating JA crosstalk with other hormone pathways. For example, we identified two miRNA/target pairs involving negative regulators of ABA signaling: Probable protein phosphatase 2C 73 and U-box domain-containing protein 19. Both genes had increased expression with JA, suggesting a dampening of the ABA pathway. This agrees with the known relationship between ABA and pathogen susceptibility, mediated by the transcription factor MYC2, which activates the ABA pathway and inhibits the JA pathway (Derksen et al., 2013). The ABA pathway contributes heavily to environmental stress tolerance (e.g. drought), so these miRNA/target interactions could modulate the trade-off between abiotic and biotic stress responses. A separate trade-off exists between stress response and growth, and JA inhibits developmental processes including seed germination and root/shoot growth (Wasternack and Feussner, 2017). We identified a miRNA/target pair comprising Auxin response factor 1, which is a repressor of auxin signaling (Ellis et al., 2005). Expression of this gene increased with JA, suggesting inhibition of auxin-associated gene expression and related growth processes. The involvement of JA-responsive miRNAs in regulating Auxin response factor genes has been reported by others, indicating that these genes represent a conserved family of targets (Bozorov et al., 2012; Xu et al., 2016; Shen et al., 2017). Finally, we identified targets involved in redox activities: Glutathione S-transferase U9 and Isocitrate dehydrogenase, which may be relevant to the ability of pokeweed to hyperaccumulate heavy metals (Peng et al., 2008; Liu et al., 2010; Zhao et al., 2012). Notably, exogenous application of JA reduces heavy metal-associated oxidative stress (Singh and Shah, 2014). Taken together, our results suggest that miRNA-mediated regulation of the JA response contributes to widespread stress resistance activities in pokeweed. We chose to study pokeweed because it is a non-model plant known to resist biotic and abiotic pressure; however, the JA-responsive miRNA/target pairs identified in this study may have agricultural relevance in terms of improving resistance to pathogen and environmental stresses in other plants.

## Data availability

Raw small RNA reads have been submitted to the SRA under project # SRP069141.

## Author contributions

KN and KH conceived the design and coordination of the study. KN performed bioinformatic analyses, statistical testing, and drafted the manuscript. AK carried out qRT-PCR validations and 5′RACE cloning. JG determined miRNA/target expression correlation and generated the interaction network. KH edited the manuscript. All authors read and approved the final manuscript.

### Conflict of interest statement

The authors declare that the research was conducted in the absence of any commercial or financial relationships that could be construed as a potential conflict of interest.

## Abbreviations

DE: differentially expressed
FC: fold change
FDR: false discovery rate
FPKM: fragments per kilobase per transcript per million mapped reads
GO: gene ontology
JA: jasmonic acid
KEGG: Kyoto encyclopedia of genes and genomes
PAP: pokeweed antiviral protein
PCC: Pearson correlation coefficient
qRT-PCR: quantitative reverse transcription PCR
RACE: rapid amplification of cDNA ends
RPM: reads per million.
