# Supplementary material for: Integration of the Pokeweed miRNA and mRNA Transcriptomes Reveals Targeting of Jasmonic Acid-Responsive Genes
Source: Front Plant Sci. 2018 May 3;9:589. doi: 10.3389/fpls.2018.00589 (PMC5944317; doi:10.3389/fpls.2018.00589)
Supplement: Supplementary file 1 [file Image_1.PDF]

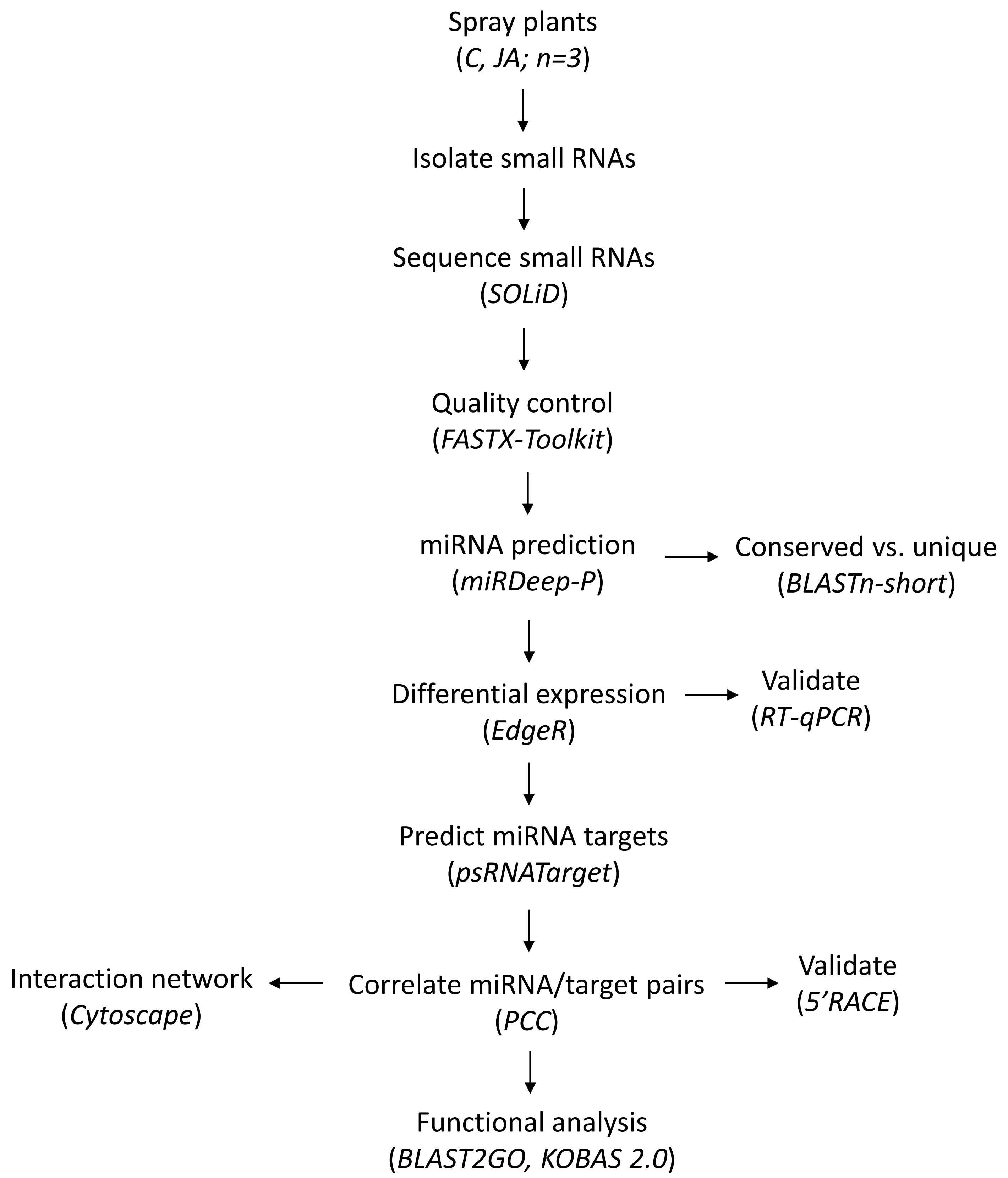

Fig. S1. Summary of the pokeweed miRNA transcriptome analysis. The applicable technique or software is indicated in brackets for each step.
