# Supplementary material for: Integration of the Pokeweed miRNA and mRNA Transcriptomes Reveals Targeting of Jasmonic Acid-Responsive Genes
Source: Front Plant Sci. 2018 May 3;9:589. doi: 10.3389/fpls.2018.00589 (PMC5944317; doi:10.3389/fpls.2018.00589)
Supplement: Supplementary file 2 [file Image_2.PDF]

A)

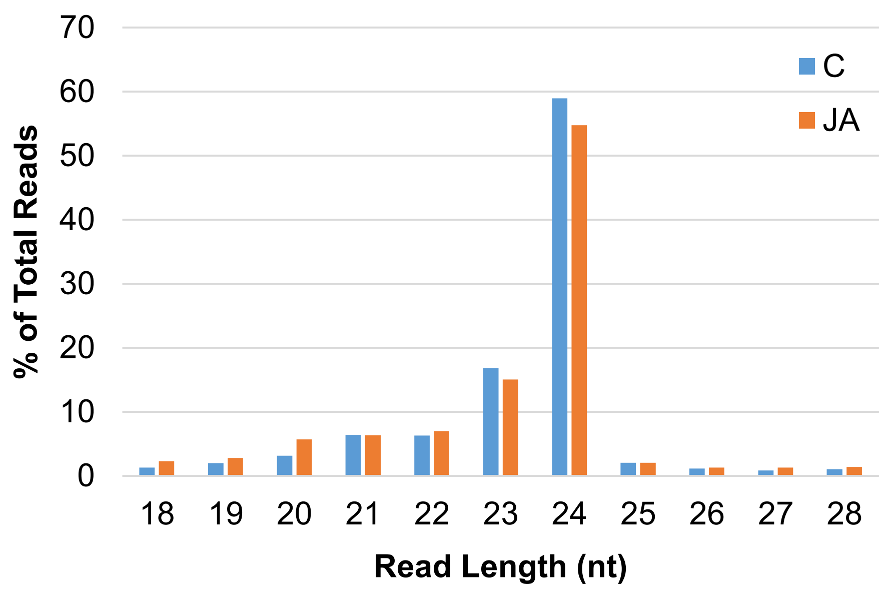

B)

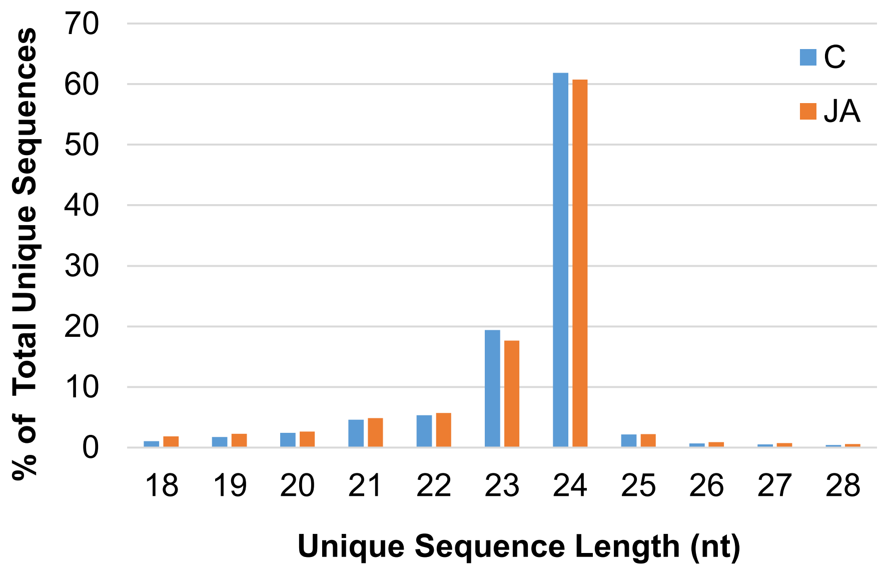

Fig. S2. The small RNA size distribution in pokeweed. Following the removal of other non-coding RNAs (rRNA, tRNA, snoRNA, snRNA), the size distribution of remaining reads was determined for A) all reads, and B) unique sequences; i.e. where reads with the same sequence have been grouped together. Values are means of three biological replicates per treatment.
