# Supplementary material for: Integration of the Pokeweed miRNA and mRNA Transcriptomes Reveals Targeting of Jasmonic Acid-Responsive Genes
Source: Front Plant Sci. 2018 May 3;9:589. doi: 10.3389/fpls.2018.00589 (PMC5944317; doi:10.3389/fpls.2018.00589)
Supplement: Supplementary file 3 [file Image_3.pdf]

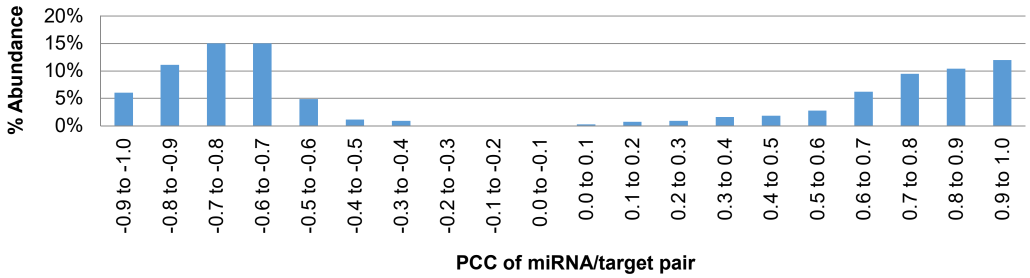

Fig. S3. Distribution of pairwise miRNA/target expression correlations. The Pearson correlation coefficient (PCC) of each miRNA/target pair was calculated based on their normalized abundances (RPM and FPKM, respectively) in control and JA samples (n = 3) and plotted as a histogram. Only differentially expressed miRNAs and targets were included in this analysis (FDR < 0.05).
