# Supplementary material for: Integration of the Pokeweed miRNA and mRNA Transcriptomes Reveals Targeting of Jasmonic Acid-Responsive Genes
Source: Front Plant Sci. 2018 May 3;9:589. doi: 10.3389/fpls.2018.00589 (PMC5944317; doi:10.3389/fpls.2018.00589)
Supplement: Supplementary file 4 [file Image_4.PDF]

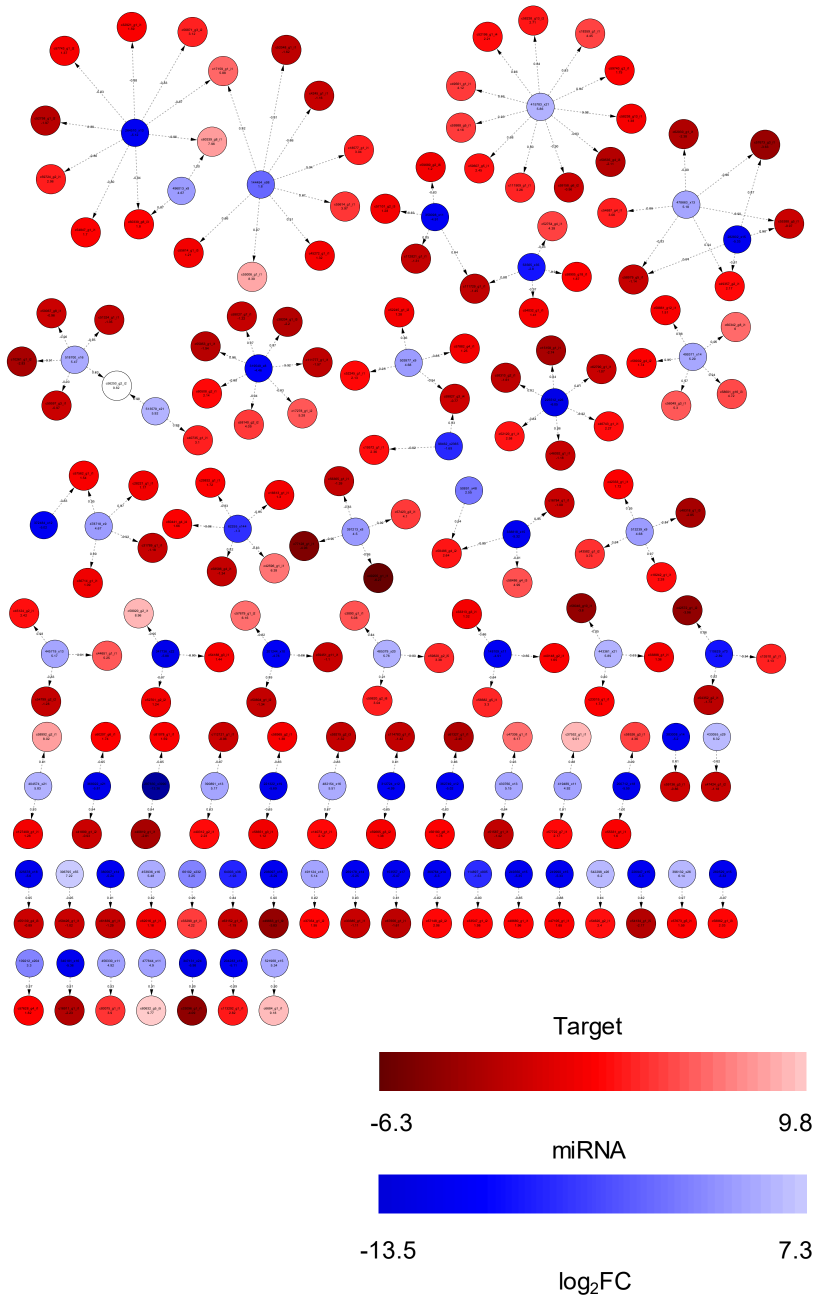

Fig. S4. Network of JA-responsive miRNA/target interactions in pokeweed. Only highly correlated miRNA/target pairs (PCC ≥ |0.8|) are included and each member of the pair is differentially expressed (FDR < 0.05). The log_2FC is indicated for each node (beneath the ID) and the PCC is indicated between nodes.
