# Supplementary material for: Integration of the Pokeweed miRNA and mRNA Transcriptomes Reveals Targeting of Jasmonic Acid-Responsive Genes
Source: Front Plant Sci. 2018 May 3;9:589. doi: 10.3389/fpls.2018.00589 (PMC5944317; doi:10.3389/fpls.2018.00589)
Supplement: Supplementary file 5 [file Image_5.PDF]

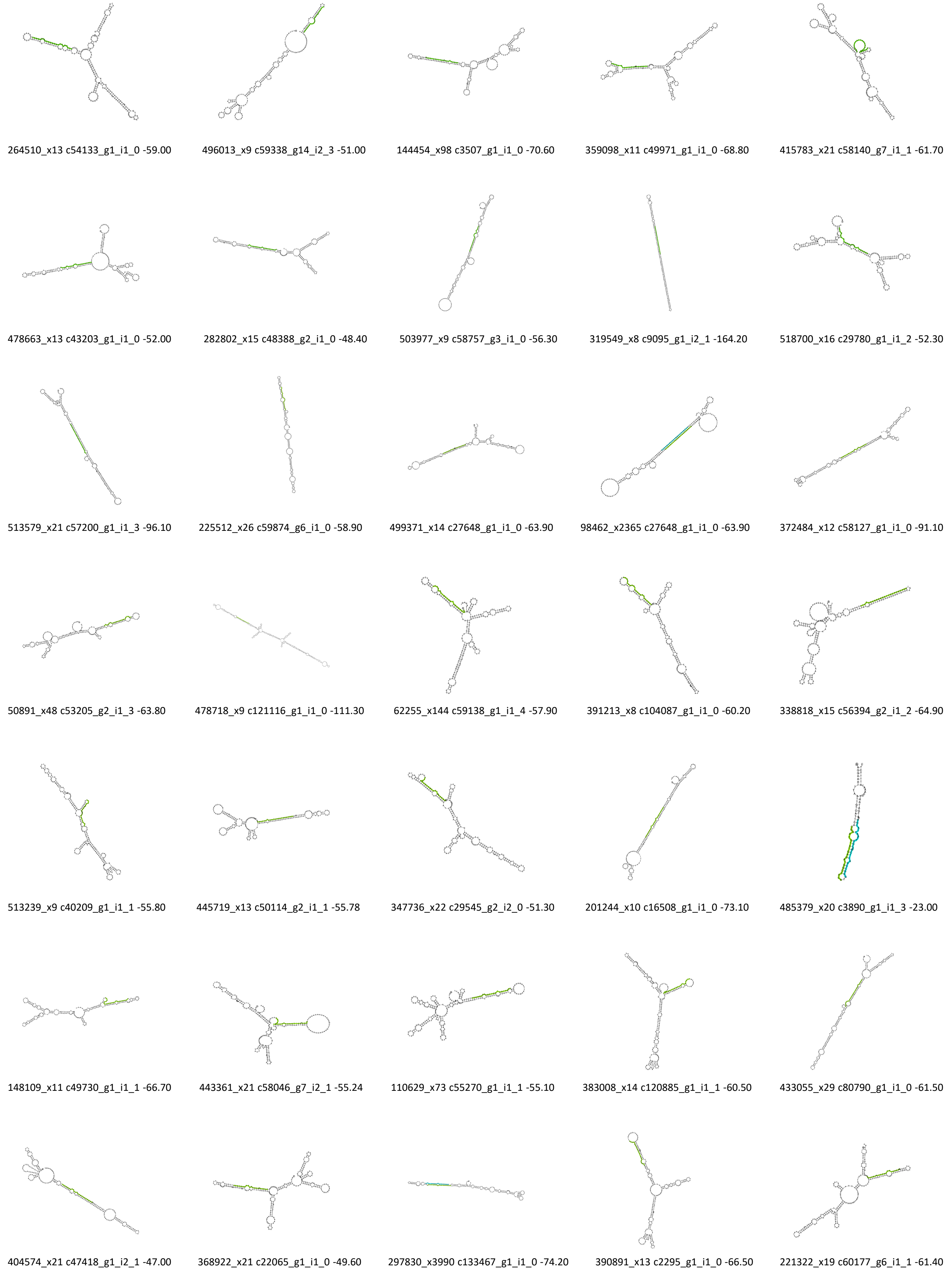

Fig. S5. The precursor structure is shown for each predicted miRNA having a highly correlated target (67 miRNAs in total). The mature miRNA sequence is highlighted in green. If a miRNA* was identified, it was highlighted in blue. For each structure, the corresponding miRNA ID, precursor mRNA ID, and minimum free energy are provided.

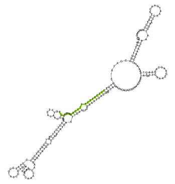
482154_x16 c114027_g1_i1_0 -45.10

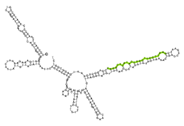
312724_x12 c31017_g2_i1_1 -67.40

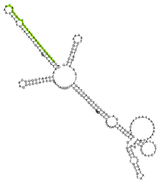
362749_x12 c55069_g3_i1_0 -72.60

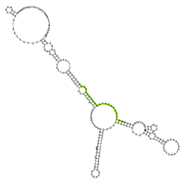
430760_x13 c57538_g3_i1_1 -64.60

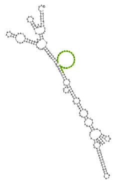
419489_x11 c25279_g1_i1_1 -52.40

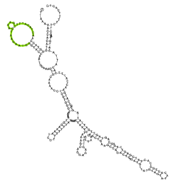
200712_x18 c33220_g1_i1_0 -47.50

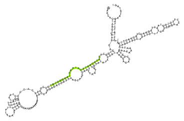
325678_x18 c57865_g6_i1_0 -57.20

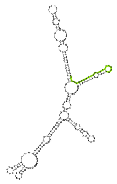
396795_x55 c59852_g1_i2_4 -58.50

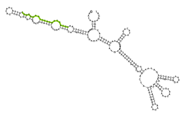
380567_x14 c36116_g1_i2_2 -58.90

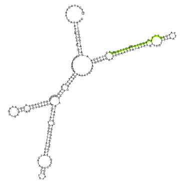
453936_x16 c57865_g5_i1_19 -55.20

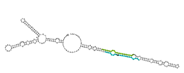
66102_x232 c29811_g1_i1_9 -58.50

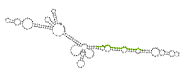
64003_x36 c55646_g2_i1_1 -48.70

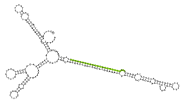
206097_x15 c58806_g5_i2_1 -76.30

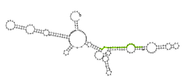
491124_x13 c14558_g1_i1_1 -57.20

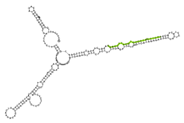
269178_x14 c60104_g8_i1_1 -72.20

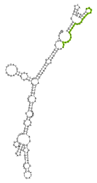
153557_x17 c154442_g1_i1_1 -51.40

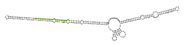
369764_x14 c16140_g1_i1_0 -90.30

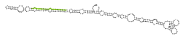
114897_x905 c133467_g1_i1_0 -74.20

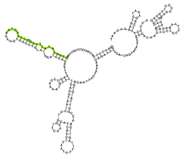
249350_x15 c57224_g3_i1_2 -45.70

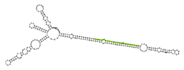
249990_x15 c59827_g3_i4_3 -84.70

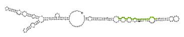
542298_x26 c127502_g1_i1_1 -49.10

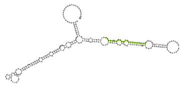
226947_x15 c47170_g1_i1_1 -62.30

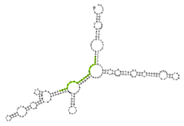
396132_x26 c49367_g2_i1_2 -49.20

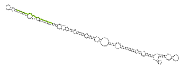
269529_x15 c60302_g1_i1_0 -65.70

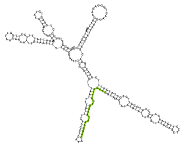
109212_x204 c59673_g1_i1_5 -50.20

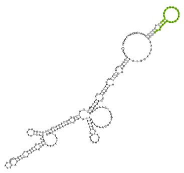
340181_x16 c54220_g1_i4_3 -50.10

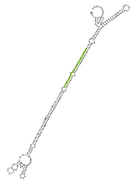
456330_x11 c80075_g1_i1_0 -75.30

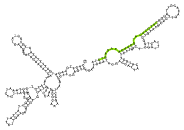
477844_x11 c136391_g1_i1_1 -57.30

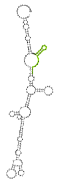
347131_x24 c60121_g7_i2_1 -51.80

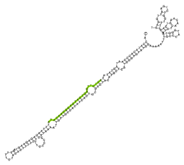
204283_x13 c139528_g1_i1_0 -72.60

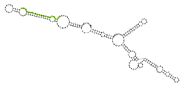
521968_x15 c111860_g1_i1_6 -49.70

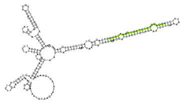
53565_x35 c59507_g15_i1_1 -79.10
